# Mechanical Stimulation: A Crucial Element of Organ-on-Chip Models

**DOI:** 10.3389/fbioe.2020.602646

**Published:** 2020-12-10

**Authors:** Clare L. Thompson, Su Fu, Hannah K. Heywood, Martin M. Knight, Stephen D. Thorpe

**Affiliations:** ^1^Centre for Predictive in vitro Models, School of Engineering and Materials Science, Queen Mary University of London, London, United Kingdom; ^2^UCD School of Medicine, UCD Conway Institute of Biomolecular and Biomedical Research, University College Dublin, Dublin, Ireland

**Keywords:** microphysiological systems, organ-on-chip, mechanobiology, biomechanics, biomechanical stimulation, pre-clinical model, tensile strain, fluid shear

## Abstract

Organ-on-chip (OOC) systems recapitulate key biological processes and responses *in vitro* exhibited by cells, tissues, and organs *in vivo*. Accordingly, these models of both health and disease hold great promise for improving fundamental research, drug development, personalized medicine, and testing of pharmaceuticals, food substances, pollutants etc. Cells within the body are exposed to biomechanical stimuli, the nature of which is tissue specific and may change with disease or injury. These biomechanical stimuli regulate cell behavior and can amplify, annul, or even reverse the response to a given biochemical cue or drug candidate. As such, the application of an appropriate physiological or pathological biomechanical environment is essential for the successful recapitulation of *in vivo* behavior in OOC models. Here we review the current range of commercially available OOC platforms which incorporate active biomechanical stimulation. We highlight recent findings demonstrating the importance of including mechanical stimuli in models used for drug development and outline emerging factors which regulate the cellular response to the biomechanical environment. We explore the incorporation of mechanical stimuli in different organ models and identify areas where further research and development is required. Challenges associated with the integration of mechanics alongside other OOC requirements including scaling to increase throughput and diagnostic imaging are discussed. In summary, compelling evidence demonstrates that the incorporation of biomechanical stimuli in these OOC or microphysiological systems is key to fully replicating *in vivo* physiology in health and disease.

## Introduction

Pre-clinical drug development requires physiologically relevant *in vitro* models which successfully recapitulate the human tissue or organ scenario *in vivo*. These predictive models are also extremely valuable for fundamental research into health and disease and for testing the response to manufactured products, food substances, toxins, pollutants, etc., providing potential as platforms for personalized medicine. Organ-on-chip (OOC) technology holds great promise in this regard as it facilitates the design of biomimetic microfluidic models incorporating multiple cell types and extracellular matrix (ECM) cues within a 2- or 3-dimensional (3D) environment, thereby replicating functional units of human tissues and organs *in vitro* (Huh et al., 2013; Caplin et al., 2015; Skardal et al., 2016; Caballero et al., 2017). These OOCs, also known as microphysiological systems, are increasingly being used to model both healthy and diseased organs and as drug screening platforms (Esch et al., 2015). This positions OOC technology as a potential route toward delivering safer and more effective treatments and unblocking the drug development pipeline which currently suffers substantial and costly attrition. As a result, there is significant interest and investment in this area from the biotech and pharmaceutical industries. Indeed, it is estimated that OOC technology could reduce pharmaceutical research and development costs by 10–26% (Franzen et al., 2019).

Organ-on-chip systems facilitate the application of multiple biochemical and biomechanical cues which direct cell behavior and ultimately replicate key aspects of tissue and organ function (Bhatia and Ingber, 2014). Biomechanical cues influence the growth and form of practically all tissues in the human body and are well established as modulators of cell signaling in health and disease (Jaalouk and Lammerding, 2009). While biomechanical stimuli can alter mass transport within tissues to modulate biochemical signaling gradients, cells can also sense these signals through mechanotransduction. Mechanobiology is the study of the cellular interpretation of biomechanical stimuli which, over the past 25 years, has become a burgeoning field of interdisciplinary research. The fundamental role of mechanobiology in many physiological processes, including the response to pharmaceuticals and other stimuli, necessitates the incorporation of biomechanical cues into OOC systems. For example, the incorporation of cyclic tensile strain mimicking lung epithelial stretch while breathing into an OOC lung-on-a-chip device is crucial to obtaining a physiological inflammatory response (Huh et al., 2010). The use of microfluidic platforms such as OOC systems for the study of mechanobiology is well established (Polacheck et al., 2013), while the innovative OOC field is producing novel approaches toward incorporating biomechanical cues into chip design as recently reviewed by Kaarj and Yoon (2019). However, to satisfy regulators and ensure reliability, OOC models with application in pre-clinical research or personalized medicine require standardized systems with validated biological models. In this review we highlight some instances where mechanical stimuli can drastically alter a given biochemical response with relevance to pre-clinical models. We summarize commercially available OOC model systems incorporating biomechanical stimuli, and review efforts to utilize these systems across different anatomical systems. The versatility of many of these OOC systems facilitates their application toward multiple organ models in addition to those validated to date, and we discuss future perspectives for this technology. As the field looks to build biological complexity in these OOC systems, it remains to be seen whether it is necessary to precisely mimic the diverse biomechanical stimuli found *in vivo*, or whether an approximation of *in vivo* biomechanics is sufficient.

## Mechanobiology and the Importance of Biomechanical Stimuli in OOC

### The Nature of Biomechanical Stimuli

**FIGURE 1:**
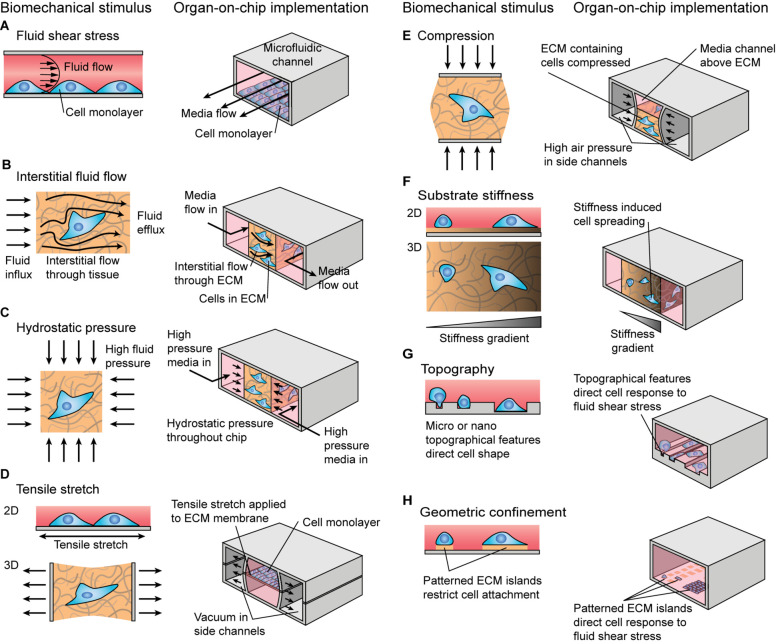
Schematic illustration of biomechanical stimuli and examples of how these stimuli could be implemented in organ-on-chip systems. **(A–E)** Active biomechanical stimuli including **(A)** fluid shear stress, **(B)** interstitial fluid flow, **(C)** hydrostatic pressure, **(D)** tensile stretch, and **(E)** compression. **(F–H)** Passive biomechanical stimuli including **(F)** substrate stiffness, **(G)** substrate topography, and **(H)** geometric confinement.

*In vivo*, cells are subjected to combinations of biomechanical and biochemical stimuli, which can interact to modulate the cellular response. Biomechanical cues are often extrinsic to the cell and can take passive or active forms. Passive biomechanical stimuli include substrate stiffness, geometric confinement, or topographic cues. Active stimuli include connective tissue tensile stretch and compression, fluid shear stress, interstitial fluid flow, and hydrostatic pressure (Figure 1).

While the nature of the biomechanical signal is important, the cellular response is also heavily dependent on the duration, magnitude, and frequency of the active biomechanical cue. Efforts to engineer connective tissues have used biomechanical signals as drivers of anabolic tissue formation and stem cell differentiation (Potier et al., 2010; Delaine-Smith and Reilly, 2012). Dynamic compression has long been applied to chondrocytes (Kim et al., 1994), with physiological loading duration, magnitude and frequency capable of eliciting anabolic responses (Anderson and Johnstone, 2017), while supraphysiological strain rates via high magnitude or frequency can drive a catabolic response (Leong et al., 2011). Experiments in fracture healing have demonstrated that mesenchymal stem cell (MSC) lineage specification can be driven *in vivo* by both magnitude and type of biomechanical signal, e.g., interstitial fluid flow or hydrostatic pressure (McMahon et al., 2008; Lee et al., 2011). These active stimuli can be further tuned to modulate stem cell differentiation through their interactions with passive stimuli such as substrate stiffness (Engler et al., 2006) and cellular confinement (McBeath et al., 2004; Gao et al., 2010).

An instance where strain magnitude and frequency can have physiological consequences is in mechanical lung ventilation, commonly used in very prematurely born infants. Children who received high frequency oscillatory ventilation as neonates had superior lung function at 11–14 years than those receiving conventional mechanical ventilation (Zivanovic et al., 2014). When the associated strain magnitudes were investigated *in vitro* using A549 alveolar analog cells, lower strain amplitudes associated with high frequency oscillatory ventilation resulted in a reduced inflammatory response which may provide an explanation for superior lung function years later (Harris et al., 2019). A great deal of literature has focused on identifying appropriate and pathological biomechanical parameters for specific cell types and tissues, and such studies involving microphysiological systems relevant to OOC models have been reviewed by Polacheck et al. (2013) and Kaarj and Yoon (2019).

### Mechanobiology Regulates Cell Behavior and Response to Pharmaceuticals

Biomechanical signals can direct cell behavior in numerous contexts. Interactions between biomechanical signals can have unexpected effects, with dynamic compression overriding the influence of hydrogel substrate to divert MSC differentiation from myogenic to chondrogenic (Thorpe et al., 2012). Biomechanical signals can also modulate the cellular response to biochemical signals including pharmaceuticals. For example, matrix rigidity can switch the functional response to the cytokine transforming growth factor-β1 (TGF-β1) in epithelial cells, with TGF-β1 inducing apoptosis in cells on soft substrates in contrast to epithelial-mesenchymal transition (EMT) in cells on rigid substrates (Leight et al., 2012). This demonstrates how changes in tissue mechanics, as often occur in disease, could confound the cellular response to a given pharmaceutical.

**FIGURE 2:**
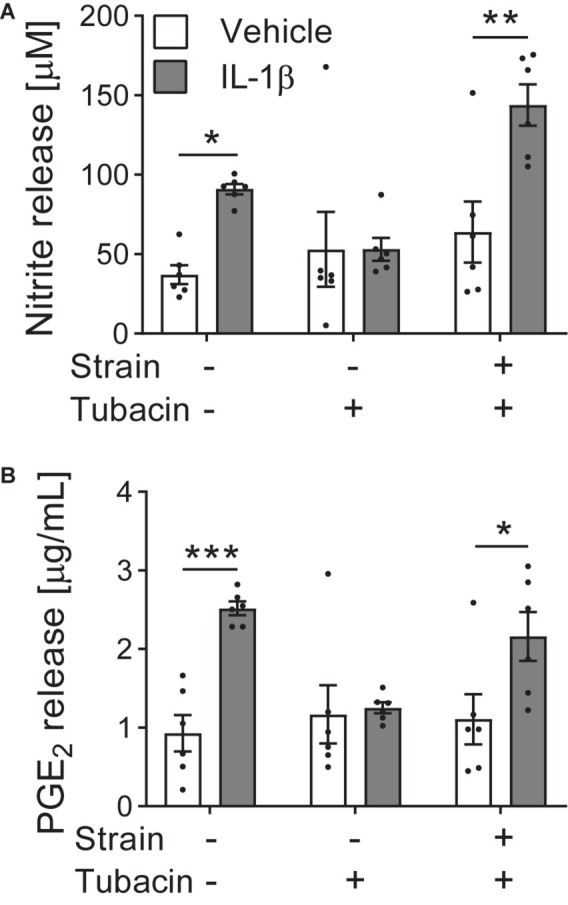
Tensile strain blocks the anti-inflammatory effects of tubacin. Bovine articular chondrocytes cultured in the presence of vehicle or interleukin-1β (IL-1β) were treated with HDAC6 inhibitor tubacin and/or cyclic tensile strain of 10% at 0.33 Hz for 24 h using a Flexcell^®^ FX5000 cell tension system. **(A)** Nitrite (nitric oxide) and **(B)** prostaglandin E_2_ (PGE_2_) release. *n* = 6; Two-way ANOVA with Sidak pairwise comparisons: *P ≤ 0.033, **P = 0.0011, ***P = 0.0004. Mean ± s.e.m. with individual values overlaid. Data adapted from Fu et al. (2019) where further details of methods can be obtained.

Active biomechanical cues can also switch the cellular response to pharmaceuticals. We have shown that dynamic tensile strain applied to cultured cells in 2D using the Flexcell^®^ system can regulate chondrocyte response to histone deacetylase 6 (HDAC6) inhibition (Fu et al., 2019). The inflammatory cytokine interleukin-1β (IL-1β) triggers nitric oxide and prostaglandin E_2_ (PGE_2_) release from articular chondrocytes, ultimately leading to the destruction of articular cartilage in disease. Tubacin, a specific inhibitor of the cytoplasmic tubulin deacetylase HDAC6, is anti-inflammatory and inhibits nitric oxide and PGE_2_ release in the absence of tensile strain (Figure 2). However, the application of biomechanical strain nullifies the anti-inflammatory effects of tubacin leading to elevated nitric oxide and PGE_2_ release (Figure 2). This compound’s efficacy as an anti-inflammatory agent is dependent on the biomechanical environment and highlights the need to include mechanical stimuli in pre-clinical testing.

The importance of biomechanical stimuli in models of the respiratory system is highlighted by studies demonstrating the ability of these stimuli to both modify disease behavior and drug responsiveness. The addition of biomechanical stimuli to an orthotopic model of human non-small cell lung carcinoma (NSCLC) decreased the sensitivity of tumor cells to tyrosine kinase therapy (Hassell et al., 2017). While this study found application of breathing motions reduced NSCLC cell proliferation and cluster formation within an Alveolus-Chip, it also resulted in the downregulation of epidermal growth factor receptor (EGFR) expression and signaling which ultimately led to the accumulation of tumor cells resistant to tyrosine kinase inhibitor mediated growth inhibition (Hassell et al., 2017). This may explain the high therapeutic resistance observed in regions of the lung which remain functionally aerated, and further demonstrates the importance of considering biomechanical stimuli in pre-clinical models.

### Intrinsic Drivers of Biomechanical Response in OOC

Mechanobiological responses are cell type dependent, and this is evident as stem cells change their mechanosensitivity with differentiation (Thorpe et al., 2010). Indeed, cellular sensitivity to mechanics may fluctuate in response to various intrinsic factors which act on timescales ranging from seconds for biomechanical memory (Heo et al., 2015), to years in the case of aging (Boers et al., 2018). Biomechanical stimuli provide integral cues to direct cell behavior, however there are several emerging intrinsic factors which influence the cellular response on the timescale of a typical study and should be considered in OOC strategies.

#### Biomechanical Memory

The response to a given biomechanical stimulus often results in changes in the cells and ECM that make up the cellular microenvironment. This in turn alters the biomechanical stimuli to which cells are exposed. The resulting mechano-reciprocal relationship between cell and biomechanical microenvironment is fundamental to tissue development and remodeling (van Helvert et al., 2018).

In addition, prior exposure to biomechanical stimuli leads to epigenetic changes determining the transcriptional response to subsequent stimuli (Downing et al., 2013; Heo et al., 2015; Li et al., 2017). Thus, the encoding of the history of biomechanical stimuli as epigenetic marks can lead to a faster more robust response upon subsequent stimulation or may temporally silence a genomic reaction to provide a refractory period of biomechanical unresponsiveness. Furthermore, different cell lineages have well documented changes in epigenetic state (Atlasi and Stunnenberg, 2017), which may impact cell behavior in response to strain.

Due to both tissue remodeling and epigenetic factors, previous exposure to biomechanical stimuli influences the response to repeat stimulation. Hence OOC systems may need to use pre-stimulated cells or precondition the model with biomechanical stimuli prior to testing the biological response to an intervention.

#### Inflammation

Chronic inflammation is associated with the deregulation of matrix signaling, tissue fibrosis and stiffening. It is well established that such changes will alter the biomechanical response of the cell and can influence a cell’s inherent biomechanical memory (Heo et al., 2015; Nowell et al., 2016). However, the direct effect of inflammatory signaling upon cellular mechanosensitivity itself is unclear. Cytokines can influence actin, focal adhesions, mechanoreceptor expression and primary cilia (Wann and Knight, 2012; Wang et al., 2018). Indeed, preconditioning of synoviocytes with inflammatory cytokines enhances mechanosensitivity (Estell et al., 2017). Therefore, it may be important to build OOC models that incorporate this interaction between inflammation and biomechanical stimuli to accurately predict *in vivo* behavior.

#### Time of Day

Cells possess an internal timing system, or circadian rhythm, allowing synchronization to predictable environmental fluctuations of the 24 h day/night cycle. This biological clock enables the temporal compartmentalization of key cell processes according to the time of day. The recent findings on Clock regulation of expression of mechanoreceptors Piezo1 and TRPV4 in bladder cells (Ihara et al., 2017), the body temperature sensitivity of TRPV4 activation (Gao et al., 2003), and diurnal actin dynamics (Hoyle et al., 2017), supports the concept of circadian fluctuations in mechanosensitivity. In addition, biomechanical stimulation, particularly in musculoskeletal tissues, may be important in regulating the clock, such that alterations in patterns of loading may disrupt clock function altering cellular behavior. Consequently, there is increasing suggestion that protocols for applying biomechanical stimuli via OOC systems should be coordinated around a physiological diurnal cycle.

#### Metabolism

Glycolysis and oxidative phosphorylation are the two major energy producing pathways in the cell. The shift between these two processes is a driving force in lineage commitment and environmental adaptation (Folmes and Terzic, 2016). Cytoskeletal remodeling and cell traction are energetically dependent upon glycolytic flux (Shiraishi et al., 2015; Hu et al., 2016), and it has recently been shown that increasing substrate stiffness leads to the downregulation of glycolysis (Park et al., 2020). Furthermore, the mechanosensor polycystin-1 has a dual function as an essential mitochondrial protein (Lin et al., 2018), together demonstrating direct connectivity of cell metabolism and mechanosensitivity. The metabolic status of cells within an OOC system will therefore influence the effect of biomechanical stimuli on regulating cell function and drug response.

## Commercial Organ-On-Chip Platforms Incorporating Biomechanical Stimuli

**FIGURE 3:**
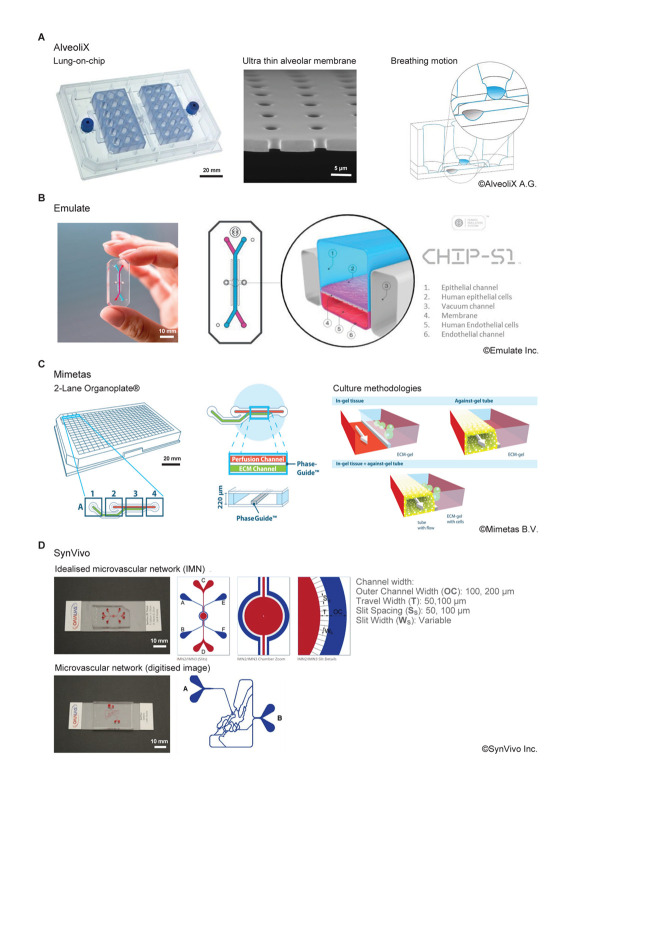
Comparison of commercially available organ-on-chip platforms incorporating a form of active biophysical stimulus. **(A)** AX12 lung chip based on a 96-well plate format, consisting of two chips supported by a plate, each of which comprise six independent units. The ultrathin membrane (blue) is deflected by negative pressure inside the basal chip chamber through an integrated micro-diaphragm (gray). Images^©^ Alveolix A.G. **(B)** The Human Emulation system from Emulate Inc. comprising organ chips which fit into the Pod^TM^ carrier. The Zoë^TM^ culture module controls the rate of flow and stretch for up to 12 chips. The Orb^TM^ provides the precise mixture of gas, power, and vacuum stretch required by the Zoë^TM^ culture module. Images^©^ Emulate Inc. **(C)** The 2-lane Organoplate^TM^ from Mimetas. Based on a 384-well plate format, it can support 96-individual models. Cells are cultured in/on ECM alongside a perfusion channel created using unique phase guide technology. Images^©^ Mimetas B.V. **(D)** SynVivo’s microfluidic chips create an idealized microvascular network to mimic the formation of, and transport across, tight and gap junctions. Networks can also be created from digitized images to replicate *in vivo* physiology more accurately. Natural tissue regions can also be incorporated within the network topology. Images^©^ SynVivo Inc.

Organ-on-chip platforms vary greatly in their design (Figure 3), however the majority of commercial systems incorporate microfluidics to supply cells with nutrients and remove waste materials, thus also providing biomechanical stimuli in the form of fluid shear and interstitial flow. A small number of systems can also stimulate cells with mechanical strain to mimic biological processes such as breathing, peristalsis or the pumping of blood through the vasculature. Other systems that apply electrical stimuli have also been developed (Feric et al., 2019). In this section we summarize several well established, commercially available OOC model systems that incorporate biomechanical stimuli (Table 1). In the subsequent section, we review efforts to validate these systems across different anatomical systems. We have focused on nine of the leading manufacturers of OOC technology who not only supply hardware but focus on the biological development of these systems.

**TABLE 1:** Commercially available organ on chip systems with integrated mechanical stimulation.

Company	Mechanical stimulation	Validated models
AlveoliX http://www.alveolix.com/	Strain: 10%, 0.16 Hz Fluid flow: passive	Alveolus: Inflammation, toxicology, fibrosis (Stucki et al., 2015, 2018; Artzy-Schnirman et al., 2019; Krempaska et al., 2020; Wang et al., 2020)
BI/OND https://www.gobiond.com/	Strain: 2.5–10%, 0–1 Hz Fluid flow: 1–300 μL/min Fluid shear: upper channel: 1.58 × 10^–6^–2.10 × 10^–4^ Pa lower channel: 0.011–9.722 Pa	Heart, midbrain organoid, cancer
CNBio https://cn-bio.com/	Fluid flow: 50–5,000 μL/min Fluid shear: dependent on model	Liver (Domansky et al., 2010; Vinci et al., 2011; Wheeler et al., 2014; Long et al., 2016; Kostrzewski et al., 2017, 2020; Sarkar et al., 2017; Rowe et al., 2018; Ortega-Prieto et al., 2019; Vacca et al., 2020). Liver/Intestine (Chen et al., 2017), Brain, Heart, Kidney, Lung, Pancreas, Skin.
Emulate, Inc. https://www.emulatebio.com/	Strain: 0–12%, 0.01–0.4 Hz Fluid Flow: 0–1,000 μL/min Fluid shear: upper channel: 0–0.009 Pa lower channel: 0–0.03 Pa	Blood-brain barrier (Sances et al., 2018; Park et al., 2019; Vatine et al., 2019) Blood vessel: Micro vessel (Jain et al., 2016) Bone marrow (Torisawa et al., 2014, 2016) Bone: Osteogenic differentiation (Sheyn et al., 2019) Lung: Small airway (Huh et al., 2010, 2013; Benam et al., 2016a, b; Jain et al., 2016; Henry et al., 2017; Si et al., 2020) Lung: Alveolus (Huh et al., 2012; Jain et al., 2018) Intestine (Kim et al., 2012; Henry et al., 2017; Villenave et al., 2017; Jalili-Firoozinezhad et al., 2018; Kasendra et al., 2018, 2020; Workman et al., 2018; Grassart et al., 2019) Kidney: Glomerulus (Musah et al., 2018) Kidney: Proximal Tubule (Jang et al., 2013; Vriend et al., 2020; Xu et al., 2020) Liver (Foster et al., 2019; Jang K. J. et al., 2019; Peel et al., 2019) Neuronal development (Sances et al., 2018)
Kirkstall https://www.kirkstall.com/	Fluid flow: 75–250 μL/min Fluid shear: dependent on model	Blood-brain barrier (Miranda-Azpiazu et al., 2018; Elbakary and Badhan, 2020) Brain: Mid brain organoids (Berger et al., 2018) Heart: cardiac tissue (Pagliari et al., 2014) Intestine (Giusti et al., 2014) Liver (Ramachandran et al., 2015; Pedersen et al., 2016; Rashidi et al., 2016; Shannahan et al., 2016) Lung (Chandorkar et al., 2017) Pancreas/Liver (Faure et al., 2016) Kidney
Micronit https://www.micronit.com/microfluidics/smart-organ-on-a-chip-platform.html	Fluid shear: 0.01–5 dyne/cm^2^	Intestine (Kulthong et al., 2020) Liver (Li et al., 2018) Pancreas (Navarro-Tableros et al., 2019; Gomez et al., 2020) Skin, Lung, Bone marrow, Neural or cardiovascular network
Mimetas https://mimetas.com/	Fluid flow: Gravity driven leveling Fluid shear: 0–0.3 Pa	Blood-brain barrier (Wilmer et al., 2016; Koo et al., 2018) Blood vessel (van Duinen et al., 2017; Beekers et al., 2018; Poussin et al., 2020) Blood vessel: Angiogenesis (van Duinen et al., 2019) Breast cancer (Lanz et al., 2017) Liver (Jang et al., 2015, 2018; Jang M. et al., 2019) Neural: CNS toxicity (Moreno et al., 2015; Wevers et al., 2018; Bolognin et al., 2019; Kane et al., 2019) Intestine (Trietsch et al., 2017; Beaurivage et al., 2019) Kidney: glomerulus (Petrosyan et al., 2019) Kidney: proximal tubule (Wilmer et al., 2016; Vormann et al., 2018; Vriend et al., 2018, 2020; Schutgens et al., 2019) Pancreas (Kramer et al., 2019) Brain: Glioma,
SynVivo https://www.synvivobio.com/	Fluid Flow: 10 nL/min–10 μL/min Fluid shear: 0.001–2 Pa	Blood-brain barrier (Prabhakarpandian et al., 2013; Deosarkar et al., 2015; Tang et al., 2018; Brown et al., 2019; Da Silva-Candal et al., 2019) Blood vessel (Silvani et al., 2019) Blood vessel: Microvascular network (Rosano et al., 2009; Prabhakarpandian et al., 2011; Lamberti et al., 2013) Cancer models (Tang et al., 2017; Terrell-Hall et al., 2017; Vu et al., 2019) Lung (Kolhar et al., 2013; Liu et al., 2019; Soroush et al., 2020)
TissUse https://www.tissuse.com/en/	Fluid shear: 0.02–2 Pa	Multi-tissue models: Intestine-Liver-Brain-Kidney (Ramme et al., 2019) Intestine-Liver-Skin-Kidney (Maschmeyer et al., 2015b) Liver-Brain (Materne et al., 2015) Liver-Intestine (Maschmeyer et al., 2015a) Liver-Kidney (Lin et al., 2020) Liver-Lung (Schimek et al., 2020) Liver-Pancreatic islets (Bauer et al., 2017)
		Liver-Skin (Wagner et al., 2013) Liver-Skin-Vasculature (Maschmeyer et al., 2015a) Liver-Testis (Baert et al., 2020) Skin-Lung cancer (Hubner et al., 2018) Single tissue models: Blood vessels (Schimek et al., 2013; Maschmeyer et al., 2015b) Blood vessels: Micro capillaries (Hasenberg et al., 2015) Bone marrow (Sieber et al., 2018) Brain (Materne et al., 2015) Hair follicle biopsies (Atac et al., 2013) Intestine (Maschmeyer et al., 2015a) Kidney (Ramme et al., 2019) Liver (Maschmeyer et al., 2015a; Materne et al., 2015; Bauer et al., 2017) Lung (Schimek et al., 2020) Pancreas: Pancreatic islets (Bauer et al., 2017) Skin (Atac et al., 2013; Maschmeyer et al., 2015a; Schimek et al., 2018) Testis (Baert et al., 2020)

### AlveoliX

The ^AX^Lung-on-Chip System is a medium throughput system which mimics the biomechanical microenvironment of the air-blood barrier of the human lung (Figure 3A). It has the footprint of a standard culture plate (127 mm × 85 mm) and comprises two chips per plate which each have six “alveolar wells.” These wells are comprised of an ultrathin (3.5 μm), porous (3 μm diameter pores) alveolar membrane. Lung cells can be cultured under air-liquid interface (ALI) on the apical side of the membrane, and endothelial cells cultured on the basal side. The membrane is cyclically deflected in three dimensions using a “micro-diaphragm” beneath the membrane which is deflected by applying negative and positive pressures through an electro-pneumatic setup. Consequently, this strain (8%, 0.25 Hz) is transferred to the alveolar membrane in a manner comparable to that observed in the human lung.

### BI/OND

The inCHIPit^TM^ offers a medium throughput organ chip with up to six cultures running in parallel. Each chip is comprised of two compartments which can be subjected to flow (1–300 μL/min) and are connected by a porous membrane. The top channel (fluid shear: 1.58 × 10^–6^–2.10 × 10^–4^ Pa) is open for added flexibility while the bottom functions as a microfluidic channel (fluid shear: 0.011–9.722 Pa) and can be subjected to cyclic strain (2.5–10%, 1 Hz). It is suitable for the cultivation of complex 3D tissues (organoids, *ex vivo* tissue, spheroids, micro tissues) as well as tissue-tissue interface models.

### CNBio

The PhysioMimix^TM^ OOC system uses open-well plates (standard footprint: 127 mm × 85 mm) which are compatible with commercial inserts, tissue-specific scaffolds and scaffold-free cultures for easy scaling and on-boarding of validated or bespoke model systems. Up to six plates can be used simultaneously to run multiple independent experiments. This system allows real-time sampling and supports long-term, automated culture, refreshing culture media at a rate of 50–5,000 μL/min with limited user input and are suitable for single-organ, two-organ, or multi-organ experiments.

### Emulate

The “Human Emulation System” platform comprises Organ-Chips, instrumentation, software, and applications to create a micro engineered system that replicates human *in vivo*-relevant physiology (Figure 3B). The Chip-S1 is composed of an elastomeric polymer, PDMS, with an upper channel (1 mm high × 1 mm wide) separated from a parallel lower channel (0.2 mm high × 1 mm wide) by a thin, flexible, porous (pore size 7 μm) membrane coated with ECM and lined with human cells. Multiple cell sources can be used including primary cells or organoids and the chips support a 3D microenvironment. The Zoë Cell Culture Module supports the culture of the chips, application of pressure-driven flow (0–1,000 μL/h, fluid shear: top channel; 0–0.009 Pa, bottom channel; 0–0.03 Pa) and stretch (0–12%, 0.01–0.4 Hz). The application of biomechanical forces (shear stress and tensile strain) and the ECM environment can be optimized for different organs or tissues to recreate a more physiological microenvironment.

### Kirkstall

The Quasi Vivo^®^ system is an advanced interconnected cell culture flow system. It is engineered to provide *in vivo* like conditions for cell growth. Available in three configurations (QV500, QV600, and QV900) this system is compatible with coverslips, membranes, barrier models, ALI culture and a range of 3D scaffolds. The Quasi Vivo^®^ system uses a peristaltic pump to create flow (75–250 μL/min).

### Micronit

Provide a range of OOC products in a variety of formats. Their core device is open and re-sealable consisting of a top and bottom layer completed by a central membrane layer. These are secured by dedicated and customizable clamps to create a cell culture platform with separately controllable fluid-flows above and below (fluid shear: 0.001–0.5 Pa). Microplate formats with active flow control systems are also offered based on fully integrated membrane valves and external actuation. Such solutions are developed and produced in a business-to-business fashion according to customer specific requirements. The open formats support sensory integration with focus applied to the optical sensing of oxygen.

### Mimetas

The OrganoPlate^®^ is a high throughput, microfluidic 3D cell culture plate capable of supporting up to 96 individual models concurrently with the footprint of a single 384-well plate (127 mm × 85 mm) and is available in 2-lane and 3-lane configurations (Figure 3C). For the 2-lane configuration, each individual model consists of two channels, a perfusion channel, and a gel channel, which are uniquely separated using phase guides rather than a physical membrane barrier. Tissues can be grown embedded in an ECM gel (lane width 375 μm) or as a perfused tubule (lane width 325 μm) against the ECM gel (surface area 1 mm^2^). Continuous gravity driven pump-free perfusion is provided using a rocker system to generate bi-directional flow (fluid shear: 0–0.3 Pa). This technology supports 3D cell culture, up to 3-layer co-culture, barrier integrity and transport, angiogenesis, and gradient formation.

### SynVivo

Microfluidic chips are functionalized to recreate complex *in vivo* like microenvironments including scale, morphology and hemodynamics along with endothelial barrier function. They can support a microvascular network that simulates the circulation inside any tissue with respect to flow, shear and pressure conditions. These microfluidic chips are available in several configurations based on the desired geometry and tissue conditions and include linear channels, bifurcating channels (comprising *in vivo* geometries), micro vascular networks (obtained from *in vivo* imaging), and idealized network designs (Figure 3D). Within the idealized network, a central chamber, flanked by vascularized micro channels, allows creation of 3D tissue OOC models for real-time studies of cellular interactions, extravasation, and drug delivery. The devices include customized micro fabricated pores to allow communication between the tissue and vascular cells while maintaining tight and gap junctions between cells. The side-by-side architecture of the chips allows for development of complex cellular morphology while maintaining real time visualization and quantitation of cell-cell and cell-drug interactions. These models are also customizable with multiple options for channel size, tissue chamber size, number of chambers, scaffolding, and barrier design. Various tissue/OOC models have been validated against *in vivo* measurements in oncology, neuroscience, and inflammation studies. A syringe pump or peristaltic pump provides continuous flow in this system (1 nL/min–100 μL/min, fluid shear: 0.001–2 Pa).

### TissUse

The HUMIMIC^TM^ platform is a miniaturized construct that closely simulates the activity of multiple human organs. The platform is available in multiple configurations and can support up to four different organ models simultaneously on a chip the size of a standard microscope slide. The organ models are supplied with media and connected by microfluidic channels (fluid shear: 0.02–2 Pa) supported by an on-chip pump. Cells and tissues can be used to emulate biological barriers as well as to grow spheroidal and matrix-supported cultures.

## Incorporation of Biomechanical Cues in OOC Models for Different Organ Systems

In the following section we outline some of the major biological systems where mechanical stimulation is well established to influence tissue function. We outline how different commercial OOC systems have been used to generate some of the most developed models of the cardiovascular, intestine, kidney and respiratory systems, and discuss how the development of the biology within these are influenced by the mechanical environment. However, the importance of mechanical stimuli to tissue development and function goes beyond these anatomical systems and will be an important component for the development of many if not all models, for example muscle, skin or fetal membrane and would similarly be expected to influence functionality and drug responses.

### Cardiovascular Models

The cardiovascular system is subject to various types and levels of biomechanical stimuli. Within the heart, highly coordinated contractions of cardiac muscle pump blood into the vessels of the circulatory system. In the vasculature, endothelial cells lining the inner surface of the blood vessels are exposed to shear stress, tensile strain, and changes in hydrostatic pressure as the result of pulsatile blood flow. These stimuli influence cell morphology, proliferation and the permeability of the vessel. Shear stress is the most significant and prominent of these forces and disruption as a result of altered flow conditions is associated with disease such as atherosclerosis, thrombosis, and aneurysm (Chiu and Chien, 2011). In straight regions of arterial trees, the vascular endothelium experiences laminar blood flow that provides high and constant pressure (>1.5 Pa). Whereas regions that branch and curve experience non-uniform, irregular, and disturbed blood flow and as such shear stress is lower (<0.4 Pa) (Gomel et al., 2018).

Key features of the cardiovascular system have been replicated using OOC technology from blood vessel-on-chip models used to study single vessels or microvasculature, to heart-on-chip models (Table 1). Drug-induced cardiotoxicity is a critical issue in drug development. However, the complex environment of mechanical and electrical stimulation means that while several heart-on-chip devices have been published, validated models using commercial systems are limited. Perhaps the most advanced of these is the Biowire II^TM^ platform from Tara Biosystems, which uses biomimetic electrical stimulation to examine the functionality of engineered cardiac tissue (Nunes et al., 2013). This heart-on-a-chip device gauges contractile activity generated by the engineered tissue as a measure of tissue function. As this review focuses on systems which directly apply mechanical stimulation, this system is not discussed here. For further review of recent developments in heart-on-a-chip models please see (Beverung et al., 2020).

Vascular components are a common feature of barrier models such as blood-brain barrier, liver, kidney, and intestine models. Disruption of the vascular barrier plays a key role in the onset and progression of several diseases, thus preventing disruption or restoring function are attractive targets for drug discovery. In the Chip-S1 from Emulate Inc., organ-specific epithelial cells are cultured within the larger upper channel, while endothelial cells are cultured in the bottom channel. These endothelial cells proliferate until a continuous channel lining is formed creating a micro-vessel that represents the supporting microvasculature of the tissue. This micro-vessel has been used to study neutrophil adhesion, rolling and intravasation in lung-chip models of asthma and chronic obstructive pulmonary disease (COPD) (Benam et al., 2016a, b). At 0.3 Pa, maximum shear stress in these systems is significantly lower than observed *in vivo*. More recently, a developmental aorta-on-chip was constructed on this platform. The shear stress values were increased to 0.5 Pa using a generic multichannel peristaltic pump, and together with cyclic membrane stretching (10%, 2 Hz) to replicate the heart rate at this stage of development (∼120 beats per minute), promoted hematopoietic stem cell formation in response to blood flow (Lundin et al., 2020).

The SynRAM^TM^ 3D inflammation model from SynVivo uses this barrier concept in a more complex chip design to study real-time rolling, adhesion, and migration processes within a microvasculature network. While simplified idealized networks can be used to reproduce constant shear and flow conditions from 0.05 to 0.4 Pa (Lamberti et al., 2013), a unique feature of this chip is that network design can also be derived from complex *in vivo* microvascular networks obtained from digitized images (Figure 3D). This produces a vascular morphology with converging and diverging bifurcations which result in varying shear and flow conditions permitting the study of inflammation and particle adhesion in a realistic and dynamic environment (Rosano et al., 2009; Prabhakarpandian et al., 2011; Lamberti et al., 2013). Indeed, the disruption to flow patterns and morphology generated near to bifurcations within the chip replicates increased adhesion of cells (platelets, leukocytes, etc.) and particles observed at these sites *in vivo* under pathological conditions (Prabhakarpandian et al., 2011).

Using OrganoPlate^®^ technology, Mimetas have created the first high throughput blood vessel-on-chip system. Human coronary artery endothelial cells seeded in the perfusion channel proliferate to cover the surface forming a micro vessel with a perfusable lumen (van Duinen et al., 2019). Fluid shear (0.16 Pa) is applied to this system in a gravity-driven manner to generate a bidirectional, oscillating flow rather than the unidirectional linear flow observed *in vivo*. Crucially, the tubular shape of this vessel only develops within the chip when adherent endothelial cells are exposed to flow thus highlighting the importance of biomechanical stimuli. This system has been used to investigate drug delivery (Beekers et al., 2018), angiogenesis (van Duinen et al., 2019) and to explore the effects of inflammation on monocyte-to-endothelium adhesion under flow (Poussin et al., 2020). In the latter, adhesion of monocytic cells to endothelial microvessels is observed in response to aerosols (Poussin et al., 2020) similar to what is observed in chip systems utilizing unidirectional flow (Benam et al., 2016b).

### Respiratory Models

Current models of the respiratory system typically focus on the alveolus or airway epithelium. As the alveolus expands and contracts during normal breathing the alveolar epithelium is exposed to cyclic tensile strain while also receiving a low level of shear stress due to air flow. Healthy epithelium typically experiences up to 12% strain, however these levels can become much higher in scarred areas which are less elastic (Waters et al., 2012). The mechanical environment of the airway itself is complex, while the contribution of strain is less, low level circumferential and longitudinal expansion and contraction will accompany normal breathing. This environment will be greatly affected under diseased conditions such as asthma where bronchoconstriction results in compressive loading of the epithelium as the result of smooth muscle activation (Tschumperlin and Drazen, 2001).

Few commercially available systems combine the ALI culture required for these models with cellular stretching to incorporate the tensile strain. The ^AX^Lung-on-a-chip, from AlveoliX is one such system (Figure 3A), it replicates the alveolar barrier whereby alveolar epithelial cells and endothelial cells are cultured in tight monolayers upon either side of a thin, porous membrane (Stucki et al., 2015, 2018). The inclusion of cyclic strain in this model (10% strain, 0.2 Hz) influences the metabolic activity of primary human pulmonary alveolar epithelial cells and increases the permeability of the epithelial barrier with no effect on cell layer integrity. Cytokine secretion by the epithelial cells is altered by the inclusion of mechanics such that interleukin-8 release is greater after 24–48 h of stretching relative to static conditions (Stucki et al., 2015).

In the Alveolus chip from Emulate Inc., human or mouse alveolar epithelial cells and endothelial cells are cultured within the Chip-S1 on opposite sides of the PDMS membrane described above (Figure 3B). The membrane is subjected to cyclic stretch to represent physiological breathing (10% strain, 0.2 Hz). This biomechanical input is essential for the replication of lung function and results in a 10-fold enhancement of the uptake of nano particulates into the alveolar epithelium over static conditions dramatically increasing reactive oxygen species production and promoting neutrophil capture and transmigration (Huh et al., 2010). Pulmonary surfactant production is enhanced within the chip further promoting epithelium integrity and barrier function while functioning as an important defense mechanism against bacterial infection (Thacker et al., 2020). Several commercial systems have been used to generate lung-on-a-chip models that mimic the complex solid and fluid microenvironment of the airway epithelium such as SynVivo’s SynALI lung model which comprises an apical channel functionalized with lung epithelial cells and surrounded by “vasculature” comprised of endothelial cells separated by a porous scaffold (Kolhar et al., 2013; Liu et al., 2019; Soroush et al., 2020). This structure allows the formation of airway tubules through ALI culture within the apical channel that transport mucus and are maintained by the surrounding endothelium (Kolhar et al., 2013; Liu et al., 2019; Soroush et al., 2020).

In the Airway-chip from Emulate Inc. primary human lung airway basal stem cells are cultured under ALI on one side of the membrane, while primary human lung endothelium is cultured on the parallel vascular channel and exposed to continuous fluid flow with a volumetric flow rate of 60 μL/h resulting in wall shear stress of 0.0017 Pa (Huh et al., 2012; Jain et al., 2018). The basal stem cells differentiate into a functional mucociliary pseudostratified epithelium containing ciliated cells, mucus-producing goblet cells, club cells, and basal cells in relevant *in vivo* proportions. The underlying human pulmonary microvascular endothelium forms a continuous cell monolayer linked by VE-cadherin containing adherens junctions. This chip accurately replicates viral infection by SARS-CoV-2 and influenza virus and can be used to study the recruitment of circulating immune cells, such as neutrophils, under dynamic flow to the site of infection. In a recent study, this chip model successfully recapitulated the effects of clinically used viral therapeutics for influenza (Si et al., 2020).

Other systems combine ALI culture with flow to generate lung models from tissue culture inserts such CNBio’s PhysioMimix^TM^ and Kirkstall’s Quasi Vivo system (Table 1). Mazzei et al. (2010) have used the Quasi Vivo system to generate co-culture models of lung epithelium and immune cells [dendritic cells (DCs), macrophages]. In the QV600 system, lung epithelium cultured on transwell inserts is subjected to ALI culture, which coupled with perfusion accelerates development of the lung epithelium with higher ciliogenesis, cilia movement, mucus-production and improved barrier function relative to static conditions (Chandorkar et al., 2017). In SynVivo’s SynALI model, epithelial and endothelial co-cultures are grown in a tubular structure to generate a central *in vitro* 3D hollow airway lumen with continuous airflow which is flanked by two *in vitro* 3D microvascular structures. The central lumen communicates via pores to these vascular channels which are cultured with living endothelium around a central lumen filled with fluid, mimicking blood flow (Liu et al., 2019).

In recent work, Si et al. (2020) demonstrated that in the Airway-chip from Emulate, only two of seven compounds identified by drug repurposing screens in 2D culture systems were effective at inhibiting viral entry of a pseudotyped SARS-CoV-2 virus. These findings in the chip have been corroborated by *in vivo* studies that similarly found one of these compounds, amodiaquine and its active metabolite (desethylamodiaquine) significantly reduced viral load in hamsters in both direct infection and animal-to-animal transmission models of native SARS-CoV-2 infection (Si et al., 2020). Thus, this study highlights the enormous potential of OOC technology to screen drug candidates more stringently prior to their use in animal studies and thus accelerate both the development and rapid repurposing of drugs.

### Intestine Models

As part of normal gut function, the intestinal epithelium is subjected to complex biomechanics. During peristalsis, waves of highly synchronized contraction move digested food through the intestinal tract deforming the intestinal mucosal layer and generating irregular compressive and tensile strains, and fluid shear stress, which vary along the length of the digestive tract as the viscosity of the digesta is altered (Brandstaeter et al., 2019). The digestive system has been another key area of focus for OOC technology as intestinal models are crucial for drug research and development, providing platforms for drug adsorption, efficacy and toxicity testing in addition to providing a range of disease models for conditions including inflammatory bowel disease and colitis.

The replication of peristaltic motions within the intestinal microenvironment is key to generation of 3D tissue architecture within organ-chips. The application of flow at 30 μL/h (0.0346 mPa) and cyclic stretch (10%, 0.15 Hz) to the human intestinal colorectal adenocarcinoma cell line (Caco-2) cultured in Emulate’s Chip-S1 (Figure 3B) promotes formation of a columnar epithelium which spontaneously grows into folds recapitulating the structure of intestinal villi (Kim et al., 2012; Grassart et al., 2019). This platform can support the co-culture of intestinal microbes for extended periods without compromising epithelial viability (Kim et al., 2012). Moreover, it is compatible with human organoid culture such that organ chips representing small intestine (Kasendra et al., 2018), adult duodenum (Kasendra et al., 2020) and colon (Sontheimer-Phelps et al., 2020) have been successfully generated in combination with co-cultures of intestine-specific endothelium. For the colon chip in particular, the use of this technology to generate a continuously perfused culture at 60 μL/h (∼0.0692 mPa) supports accumulation of a mucus bilayer with impenetrable and penetrable layers, and a thickness similar to that observed in the human colon which can be analyzed non-invasively in real time (Sontheimer-Phelps et al., 2020).

In Micronit’s gut-on-chip model, Caco-2 cells are subjected to flow at 100 μL/h producing a shear stress of ∼0.02–0.17 mPa at the cell surface (Kulthong et al., 2020). Following culture in this manner for 21 days, caco-2 cells form a continuous epithelium with greater height than equivalent transwell cultures that have an enhanced barrier function. Caco-2 cell differentiation was comparable with static cultures in this model (Kulthong et al., 2020).

In Mimetas’ 3-lane Organoplate^®^, perfused intestinal tubules can be cultured in a high throughput manner as a mono-culture or co-culture with immune cells or blood vessels and stromal tissue to generate a model of the intestinal epithelium (Trietsch et al., 2017). The inclusion of stromal tissue interactions is an important factor for replicating physiological cellular interactions. In this system, Trietsch et al. (2017) cultured Caco-2 cells against an ECM gel, which proliferate upon application of bi-directional flow to form a confluent tube. Perfusion is critical for tubule formation. Beaurivage et al. (2019) have recently used this model to mimic the effects of *Escherichia coli*-activated DCs on the intestinal epithelium. Through the addition of a cytokine cocktail of IL-1β, tumor necrosis factor α (TNF-α) and interferon-γ (IFN-γ), they were able to replicate the loss of barrier function observed in irritable bowel disease (Beaurivage et al., 2019). Similar results were achieved using iPSCs which can be induced to undergo differentiation within the Organoplate^®^ to express mature intestinal markers, including markers for Paneth cells, enterocytes and neuroendocrine cells (Naumovska et al., 2020).

In addition to drug efficacy, biomechanics can also mediate pathogen infectivity. In the small intestine, the incorporation of both fluid flow (30 μL/h, fluid shear: 0.346 mPa) and tensile strain (10%, 0.15 Hz) mimicking peristalsis promotes formation of a more physiologically relevant 3D architecture. Grassart et al. (2019) observed that these mechanically active intestine chip cultures better replicate bacterial infection uncovering a mechanism whereby *Shigella flexneri* exploits the epithelial crypt microarchitecture and active biomechanics to efficiently invade the intestine. In the region of 70% of all drugs are administered orally (Brandstaeter et al., 2019). Their processing and effectiveness depend crucially on gastric mechanics thus the incorporation of active biomechanical stimuli into OOC gut models is essential to the successful use of this technology.

### Kidney Models

Several OOC models have focused on the kidney proximal tubule as the site at which active clearance, reabsorption, intracellular concentration, and local interstitial accumulation of drugs primarily occurs. The epithelium in this region is continually exposed to shear stress in the region of 0.02 Pa as the result of constant flow of the glomerular filtrate which influences cell morphology causing alignment and elongation of kidney epithelial cells in the direction of flow (Vriend et al., 2020), and modulates expression of apical and basolateral transporters and sodium transport (Duan et al., 2010).

Nephrotoxicity is a major cause of drug attrition during pre-clinical pharmaceutical development and is responsible for almost 20% of failures during Phase 3 clinical trials underlying the limitation of current methodologies (i.e., 2D cell culture and animal models) to predict the human response. The inclusion of biomechanical stimuli in kidney models examining drug toxicity is essential as exemplified by the use of both the OrganoPlate^®^ (Mimetas) and Chip-S1 (Emulate) systems which show that both albumin uptake and drug efflux are enhanced in response to fluid shear stress despite the differential use of uni-directional and bi-directional flow in these models (Jang et al., 2013; Vriend et al., 2020). Jang et al. (2013) report that cisplatin toxicity more closely replicates the *in vivo* response observed than traditional culture techniques. Thus, the use of these models which incorporate biomechanics to screen for kidney injury early in the drug discovery process will provide greater capacity to predict responses in humans.

### Musculoskeletal Models

The nature and magnitude of biomechanical stimuli experienced by the musculoskeletal system are highly varied both between connective tissue types and within them due to the extreme forces that must be endured during physical activity. These mechanical stimuli are essential to the health and maintenance of the tissue; regulating cell morphology, proliferation matrix production and catabolism. Articular cartilage is routinely exposed to diverse mechanical stimuli consisting of compressive, shear and tensile strains as well as associated alterations in fluid shear and osmolality as a result of normal physical activity (Knecht et al., 2006). Energy-storing tendons like the Achilles are designed to stretch and recoil to increase efficiency during locomotion and are thus subjected to high magnitude stresses and strains during exercise. In the bone, oscillatory fluid flow generated by compressive loading generates shear stress in the lacunar-canalicular network which influences both the maintenance and healing of bone tissue and is essential for bone health. Inappropriate cellular responses to these stimuli result in the disruption of tissue homeostasis and can lead to conditions such as tendinopathy and osteoarthritis (Felson, 2013; Dean et al., 2017). Thus, the incorporation of biomechanical stimuli into OOC models of the musculoskeletal system is essential. However, few commercial systems have been used to develop models in this field to date.

Existing models from Emulate, Micronit and TissUse have focused on bone or bone marrow while models for muscle, cartilage or tendon are overlooked. This is likely due to the difficulty in replicating the complex architecture of these tissues and the more dynamic biomechanical environment the cells experience. A bone-on-a-chip generated using Emulate’s Chip-S1 (Figure 3B) utilizes an inducible, MSC-bone morphogenic protein-2 (BMP-2) overexpression system cultured on one side of the membrane to examine osteogenic differentiation under flow (Sheyn et al., 2019). In this system, cells grown on Bone-chips under flow (30 μL/h, shear stress: 0.346 mPa) showed enhanced survival and proliferation relative to cells grown under static conditions. MSCs cultured in this manner exhibited greater expression of the osteogenic markers osteopontin, bone sialo-protein, and collagen type I, despite the use of constant flow in this system (Sheyn et al., 2019).

Hydroxyapatite coated zirconium oxide scaffolds incorporated within the multiorgan chip (MOC) from TissUse combine tissue engineering techniques with OOC technology and have been used to generate bone-marrow-on-chip (Sieber et al., 2018). In this model, a cell seeded scaffold of MSC and multipotent haematopoietic stem and progenitor cells (HSPC) is cultured within the MOC which comprises two separate independent channel circuits, each hosting a single culture compartment interconnected by the channel system. One of these compartments is used for the cell scaffold, while the other functions as a medium reservoir. The flow rate (5 μL/min) is controlled by an on-chip peristaltic micro pump (frequency of 2 Hz for continuous dynamic operation) integrated into each circuit (Sieber et al., 2018). Long term culture of HSPCs is maintained in the MOC for a period of 28 days. The cells form a microenvironment reminiscent of the *in vivo* bone marrow niche within the scaffold, and remain in their primitive, undifferentiated state (Sieber et al., 2018).

## Perspectives

### Challenges Associated With Incorporating Biomechanical Stimuli in OOC Models

Incorporating appropriate physiologically representative biomechanical stimuli into OOC models is challenging. A major confounding factor in the replication of *in vivo* biology is tissue structure. Cells within their native environment can withstand significantly higher levels of mechanical stimulation due to the structural organization of the tissue. For example, joint loading during physical activity subjects articular cartilage to forces several times greater than body weight. However the unique anisotropic structure of this tissue (depth dependent variation in collagen fibril alignment and confinement of hydrated proteoglycan) allows these forces to be dissipated such that the cellular strains experienced by chondrocytes are actually much lower (Bergmann et al., 1993). Thus, further integration of OOC technology with tissue engineering techniques such as 3D bioprinting to achieve structurally aligned matrices or generate 3D scaffolds within chips, has the potential to create better representations of the mechanical environment within organ models. This could unlock new application areas for OOC, such as modeling fibrosis and tumor stroma.

The successful recapitulation of the *in vivo* biomechanical environment will also be dependent upon the ability of organ chips to incorporate multiple forms of mechanical stimuli within a single device. Thus far commercial systems have achieved the integration of stretch and flow (Emulate Inc., BI/OND; Figures 1A,B,D). However, to date no commercial systems are available which apply compression in organ chips. The incorporation of compressive strain into OOC systems will be essential to the development of multiple models particularly those mimicking the musculoskeletal system. Topography and geometric confinement (Figures 1G,H) provide two biomechanical cues which while not yet incorporated into the above reviewed commercial systems, could be easily integrated into future iterations of these to build biomechanical complexity. Moreover, integrating these stimuli into a single model presents significant technical challenges both in terms of the interactive effects these stimuli will exert upon each other (e.g., flow rate changes as the result of stretch induced changes in channel volume and shape) and the increased complexity of the biological outcome. To replicate the body’s fluid shear conditions more accurately, organ chips should feature greater ranges of fluid shear levels and more variable types of flow such as oscillatory or pulsatile flow. Once again, the successful integration of these stimuli will enhance the accuracy of organ models and thus better replicate tissue function in health and disease.

A caveat to increasing the biological complexity of these models is the conflict this represents with analytical requirements such as real-time imaging, sampling, and scaling to increase throughput. Moreover, more complex models will likely produce more complex outputs due to additional cellular and matrix interactions which could be difficult to interpret.

One of the many advantages of OOC systems are that they have the potential to be accessible in ways that cannot easily be achieved *in vivo*. Thus, the ability to culture cells for extended periods with regular sampling of culture media and cellular by-products are a necessary feature. Mechanical stimuli within OOC systems could also be used more generally to improve mechanobiological studies. Therefore, researchers must be able to monitor both the cells and their responses in real-time in a non-destructive manner using existing research methodologies for this technology to be adopted more readily. At the same time, for these systems to be successful within the pharmaceutical industry, they need to be high throughput and have the potential for significant automation of downstream analyses. This undoubtedly presents significant technical challenges relating to the development of robust standardized equipment supporting these organ chips for the application of multiple types of stimuli at consistent, and tightly controlled levels.

The use of OOC technology for diagnostic approaches or to deliver personalized medicine is highly attractive. The incorporation of this technology into clinical practice to determine drug responsiveness of patient samples and inform clinical decisions means that these models must be established in a highly standardized manner and subjected to well-defined mechanical input to deliver clear outcomes.

### How Much Mechanobiology Do We Need?

Organ-on-chip models incorporating active mechanical stimuli have arrived at similar findings demonstrating the crucial role of biomechanics in replicating *in vivo* behavior and dictating drug response. In this review, we identify several studies demonstrating that incorporation these stimuli into OOC models has the potential to create more sophisticated organ models that better represent the *in vivo* scenario. In vascular models, appropriate modulation of inflammatory responses is observed at shear stress levels far below those found *in vivo* (Benam et al., 2016a, b; Poussin et al., 2020). In the airway, stretch influences viral entry and drug efficacy (Si et al., 2020). In the kidney, the application of apical shear stress modulates drug uptake and nephrotoxicity (Duan et al., 2010; Vriend et al., 2020). While in the intestine, the inclusion of peristalsis-like stretch creates a more accurate representation of bacterial infection (Grassart et al., 2019).

These examples from different organ systems provide strong evidence to support the consideration of mechanobiology when designing *in vitro* OOC model systems to obtain a more accurate, reliable prediction of the human response prior to clinical trials. Ultimately, this will accelerate the drug development process by both identifying potential drug candidates earlier in the pipeline and by ruling out many of those that will fail in subsequent clinical trials. However, none of these models precisely mimic the entire *in vivo* biomechanical environment, rather focusing on a key stimulus delivered at approximately physiological intensities. These systems also do not account for pathological patient-specific biomechanics which typically deviate from what is considered physiologically normal and may be important in the use of OOC for personalized medicine. This begs the question, how accurately do biomechanical stimuli need to be replicated? Several commercial systems already incorporate a sufficient level of biomechanical stimulation to modulate drug efficacy in multiple organ models and are beginning to be adopted by the pharmaceutical industry in their current forms. The requirement for standardized systems suggests future research should focus on developing the biology within these existing systems. Validation of these OOC systems in a clinical context requires the replication of *in vivo* human biology, which must provide the benchmark when considering the extent to which mechanical stimuli should be incorporated. A greater understanding of how individual forms of biomechanical stimuli influence cell behavior in both health and disease is therefore required to develop these models and enhance their ability to predict drug performance in humans.

## Author Contributions

CT, HH, and ST reviewed and evaluated the literature and drafted the manuscript. MK was involved in the conception of the review and critically revised the manuscript. SF provided preclinical data. All authors contributed to manuscript revision and approved the submitted version.

## Conflict of Interest

MK is Director of the Queen Mary + Emulate Organs-on-Chips Centre and CT is the centre scientist of the Queen Mary + Emulate Organs-on-Chips Centre which is part funded by Emulate Inc. Emulate Inc. were not involved in the preparation of this review other than the provision of data on device specifications as provided by all featured manufacturers. The remaining authors declare that the research was conducted in the absence of any commercial or financial relationships that could be construed as a potential conflict of interest.
