# Corrigendum: Mechanical Stimulation: A Crucial Element of Organ-on-Chip Models

**DOI:** 10.3389/fbioe.2021.658873

**Published:** 2021-02-18

**Authors:** Clare L. Thompson, Su Fu, Hannah K. Heywood, Martin M. Knight, Stephen D. Thorpe

**Affiliations:** ^1^Centre for Predictive in vitro Models, School of Engineering and Materials Science, Queen Mary University of London, London, United Kingdom; ^2^UCD School of Medicine, UCD Conway Institute of Biomolecular and Biomedical Research, University College Dublin, Dublin, Ireland

**Keywords:** microphysiological systems, organ-on-chip, mechanobiology, biomechanics, biomechanical stimulation, pre-clinical model, tensile strain, fluid shear

Hannah K. Heywood was not included as an author in the published article. The corrected Author Contributions Statement appears below.

CT, HH, and ST reviewed and evaluated the literature and drafted the manuscript. MK was involved in the conception of the review and critically revised the manuscript. SF provided pre-clinical data. All authors contributed to manuscript revision and approved the submitted version.

The authors apologize for this error and state that this does not change the scientific conclusions of the article in any way. The original article has been updated.

